# Commensal microbes and p53 in cancer progression

**DOI:** 10.1186/s13062-020-00281-4

**Published:** 2020-11-19

**Authors:** Ivana Celardo, Gerry Melino, Ivano Amelio

**Affiliations:** 1 MRC Toxicology Unit, University of Cambridge, Cambridge, UK; 2 Department of Experimental Medicine, TOR, University of Rome Tor Vergata, Rome, Italy; 3 School of Life Sciences, University of Nottingham, Nottingham, UK

**Keywords:** Microbiota, p53, Tumour suppression, Oncogenes, Microenvironment

## Abstract

Aetiogenesis of cancer has not been fully determined. Recent advances have clearly defined a role for microenvironmental factors in cancer progression and initiation; in this context, microbiome has recently emerged with a number of reported correlative and causative links implicating alterations of commensal microbes in tumorigenesis. Bacteria appear to have the potential to directly alter physiological pathways of host cells and in specific circumstances, such as the mutation of the tumour suppressive factor p53, they can also directly switch the function of a gene from oncosuppressive to oncogenic. In this minireview, we report a number of examples on how commensal microbes alter the host cell biology, affecting the oncogenic process. We then discuss more in detail how interaction with the gut microbiome can affect the function of p53 mutant in the intestinal tumorigenesis.

## Background

In addition to the genetic factors [1], microenvironmental components certainly influence cancer progression and initiation, as clear evidence emerged on the contribution of integration of extrinsic and intrinsic factors in the disease pathogenesis [2, 3]. Genomic studies have clarified the genetic basis for several malignancies [4–6], as for examples neuroblastoma, which shows a clear pattern of mutations with a well-defined prognostic value [7–11]. In a wider perspective, however, distal interaction among different organs can also contribute to pathogenesis of cancer. The gut microbiota has emerged as determinants not only for the health of gastrointestinal tract (GI), but also for distal districts such as brain, pancreas and liver [12–15]. Causative links between dysbiosis and neurodegeneration, diabetic, obesity and cancer have been postulated and in part also demonstrated [16–19].

Colorectal cancer (CRC) is directly linked to gut microbiota. CRC-enriched bacteria have been identified with faecal metagenomic approaches on patients with CRC [20, 21]. These include *Parvimonas micra, Fusobacterium nucleatum, Bacteroides fragilis, Porphyromonas asaccharolytica, Thermanaerovibrio acidaminovorans, Prevotella intermedia and Alistipes finegoldii* [22, 23]. They may serve as diagnostic markers of diseases; they may also direct on a better understanding of the CRC pathogenesis and may provide therapeutic strategies in the future.

An important aspect that links dysbiosis and cancer pathogenesis is inflammation. Bacterial infection can indeed result in cancer. Gastric infection with *Helicobacter pylori* causes persistent inflammation and gastritis, resulting in a significant proportion of individuals in stomach malignancies [24]. Similarly colitis can result in tumorigenesis [25, 26, 27]. The microbiota is altered by the colitis-associated inflammation as well as altered microbiota might be causative of colitis. Interaction with host and commensal bacteria seems to have crucial implications on human health. There is indeed a fine balance between microbiota, inflammatory response and immune system that influences tumorigenesis.

Here, we will summarise major recent advance on the interaction between microbiota and human malignancies, with a particular focus on GI conditions. We will also discuss the potential paradoxical impact that microbiota can have on tumour suppressive mechanisms, such as the recently reported effect of switching mutant p53 from tumour-suppressive to oncogenic role [28].

## The microbiota in Cancer progression

**Fig. 1:**
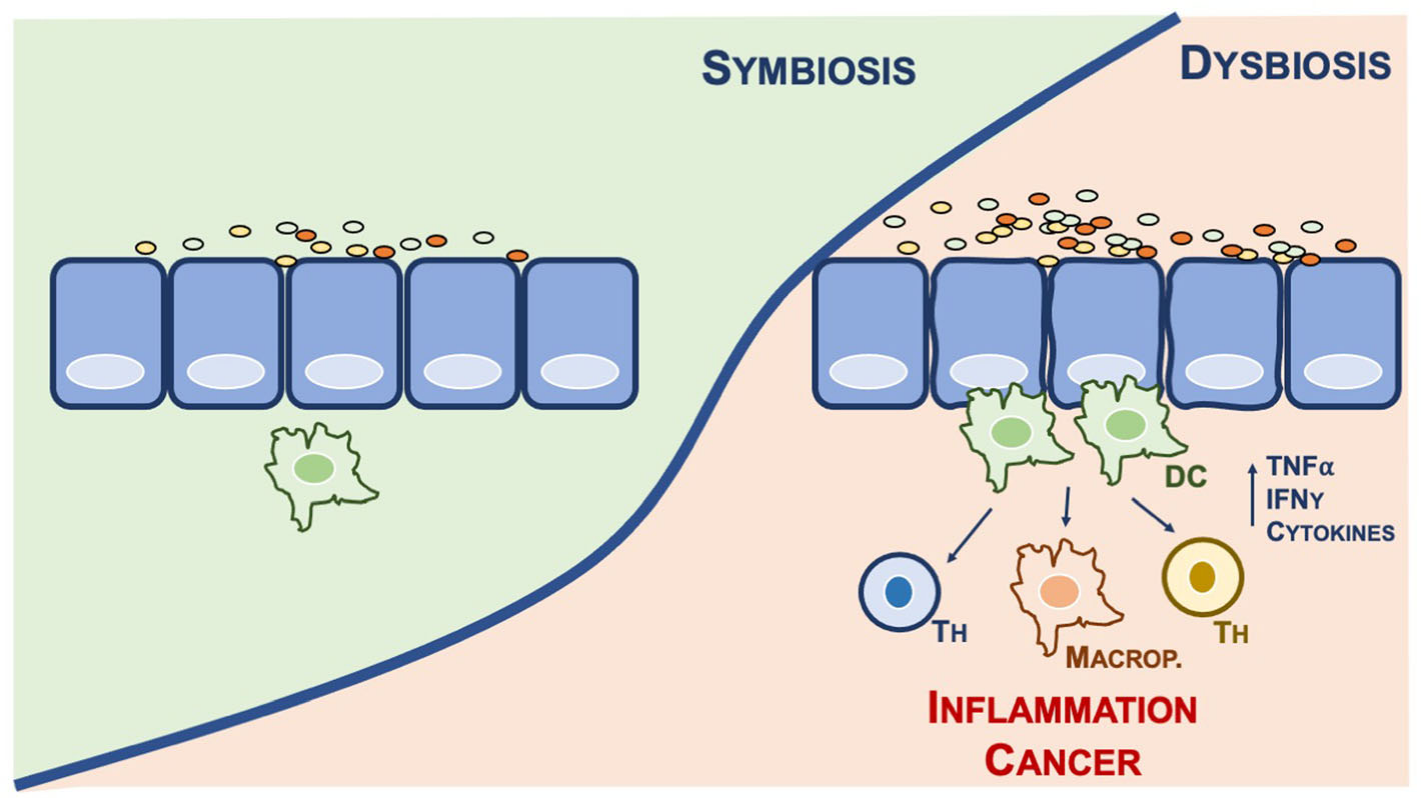
Gut microbiota symbiosis vs dysbiosis. Alterations of the fine balance within commensal bacteria colonies in the gut microbiota can affect immune systems with a consequence release of pro-inflammatory cytokines and mediators, such as TNF-a and IFN-g. Recruitment of immune system can lead to sustained inflammation, which has been associated to increased susceptibility to cancer. DC, Dendritic Cells; Th, T helper cells; Marcop, Macrophages

Dysbiosis is generally associated to commensal microbes outcompeted [29]; this can favour establishment of pathogenic microbes that might have causative roles in cancer (Fig. 1). Antibiotics can be the cause of outcompeting gut commensal microbiome, thus facilitating establishment of pathogenic bacteria colonies that ultimately may lead to inflammation and in specific circumstances to cancer. A deep understanding of the consequences of gut dysbiosis and the molecular basis of these represent a priority to direct research efforts in the area of cancer pathogenesis [30].

Several bacteria strains have been identified [31, 32] and associated to cancer progression and in some instance, these can produce directly or indirectly DNA damage. *Fusobacterium nucleatum* has an established role in gastric, pancreatic and colorectal cancers [33–35]. Interaction between FadA adhesin protein from *F. nucleatum* can interact with E-Cadherin, triggering activation of beta-catenin signalling, which ultimately modulates inflammatory and oncogenic responses to promote tumorigenesis. CRC patients display an elevated level of FadA protein and Wnt7b and NF*k*B2 mRNAs. Thus, FadA appears to promote tumorigenesis and *F. nucleatum* associated inflammation [36]. FadA has also been implicated in the control of natural killer (NK) cell cytotoxicity via its interaction with T cell immunoglobulin and ITIM domain (TIGIT), producing cell death in human lymphocytes [37, 38]. Also *Citrobacter* can mediate Wnt-β-catenin activation through R-spondin 2 in infected mice, producing expansion of intestinal stem cells and poor differentiation [39]. *Enterotoxigenic Bacteroides fragilis* also displays the ability to promote tumorigenesis in CRC mouse model by stimulating exaggerated immune responses via T helper 17 (Th17) cells [40]. Interaction between microbes and immune system is therefore central in the mechanisms underlying disease onset and progression.

Similarly, to *Fusobacterium nucleatum,* also *P. anaerobius* has been associated to CRC*. P. anaerobius* displays interaction ability with α2/β1 integrin expressed on colonic cancer cells by its surface protein, putative cell wall binding repeat 2 (PCWBR2). Interaction occurs on the CRC cells, but not with the normal colonic epithelial cells. α2/β1 integrin in turn activates PI3K-Akt pathway, which promotes cell proliferation, as well as inflammation via nuclear factor kappa-light-chain-enhancer of activated B cells (NF-κB), recruiting in the tumour microenvironment the tumour-infiltrating MDSCs and TAMs [41]. *P. anaerobius* can also interact with toll-like receptor 2 (TLR2) and TLR4 on colon cells, and modulate myeloid-derived suppressor cells (MDSCs), granulocytic tumour-associated neutrophils and tumour-associated macrophages (TAMs), thus promoting CRC [42]. In the context of this wide interplay governing the balance between microbes, colon epithelia and immune system it becomes central the response to xenobiotics. Within these, it is of particular relevance the chemotherapy and the general responsiveness to anti-cancer therapy. Hence, efforts should be directed to examining the effect of bacterial species on chemotherapies and cancer immunotherapies and how this interplay influence the response to cancer therapy.

## The microbiota in Cancer therapy

Prediction on how the gut microbiota may influence the therapeutic response to anticancer agents should take into account a myriad of intrinsic and extrinsic factors and their own interaction with each other. A large part of the altered anticancer therapy response is associated to the effect on the immune system; hence this indicates that these aspects are of significant importance for immunocheckpoint blockade therapy. However, gut microbiota has been proved to equally affect and alter response to standard anti-cancer therapy, with mechanisms that can involve or not the host immune system.

Radiotherapy represents an established curative and palliative therapeutic protocol for different types of cancers. A significant part of radiotherapy efficacy is mediated by potent immune modulatory effects, including tumour-associated antigen cross-priming with antitumor CD8^+^ T cell elicitation and abscopal effects. Vancomycin, a gram-positive bacteria effective antibiotic, is capable of potentiating the radiotherapy-induced immune response against the tumour, thus inhibiting tumour growth in mouse transplanted B16-OVA melanoma and TC-1 lung/cervical models. Mechanistically, the effect was mediated by elicitation of cytolytic CD8+ T cells and IFN-γ response. Hence, depletion of vancomycin-sensitive bacteria is proposed to enhance the antitumor activity of radiotherapy [43].

*Enterococcus hirae* and *Barnesiella intestinihominis* appears involved in response to the anti-cancer immunomodulatory agent cyclophosphamide. In particular *B. intestinihominis* shows ability to promote the infiltration of IFN-γ-producing γδT cells in cancer lesions, while *E. hirae* increases the intratumoral CD8/Treg ratio by translocating from the small intestine to secondary lymphoid organs. The bioactivity of these microbes as well as the response to cyclophosphamide can be limited by the immune sensor NOD2 [44]. Memory Th1 immune cells promoted by *E. hirae* and *B. intestinihominis* showed ability to predict longer progression-free survival in patients treated with chemo-immunotherapy. *Clostridium* has been proven to be an important player in the regulation of bile acids, thus influencing chemokine CXCL16 release. Bile acids can influence production by liver sinusoidal endothelial cells of the CXCL16, which recruits natural killer T (NKT) cells to the tumour, inhibiting primary and metastatic tumour growth [45].

Effects of microbiome on chemotherapeutic response not associated to immunomodulation has also been shown. The chemotherapeutic drug gemcitabine (2′,2′-difluorodeoxycytidine), first choice for treatment of several malignancies, including pancreatic adenocarcinoma, can be metabolized by bacteria into its inactive form, 2′,2′-difluorodeoxyuridine. Intratumor *Gammaproteobacteria* was able to promote gemcitabine resistance in a mouse model of colon cancer. Consistently a significant proportion of human PDACs (76%) were found positive to bacteria [46]. Hence, resistance to gemcitabine treatment, that currently emerges in PDAC treatment might be associated to altered microbiome [47].

## p53 mutations in cancer pathogenesis

Sporadic mutations in p53 are observed more than 50% of all human cancers, while germline p53 mutations that abolish its function show a high predisposition to tumour formation in a syndrome known as Li-Fraumeni [48]. The pattern of mutations in p53 gene is very peculiar: mutations occur in the largest majority of cases as missense, leading to expression of mutant proteins. Only a small proportion (less than 10%) results in non-sense (earlier stop codon) or deletion of the gene [49].

The canonical signalling promoted by p53 results in three major biological response: growth arrest, DNA repair and eventually apoptosis [50–54]. The arrest of the cell cycle leads to a temporary arrest of the proliferation, which prevents replication of damaged DNA and the transfer to daughter cells. The p53-mediated cell cycle arrest is mediated by the transcriptional activation of p21 [55–59], which is then followed by upregulation of a large number of pro-apoptotic genes, including Puma, Noxa, Bad, Bax, Bak, p53AIP1, and Fas [57, 60–63]. The promotion of DNA repair occurs in the time-frame between cell cycle arrest and apotptosis [64–68]; if DNA repair is successful, the cell cycle resumes.

p53 neomorphic proteins [49, 69–71], associated to mutations in p53 sequence, were implicated in alteration of physiological cellular signalling, including the function of the p53 family members, p63 [72–76] and p73 [77–81] and other transcriptional factors, such as HIF-1 [82] in several different cancer types [83–85]. p63 and p73 indeed in addition to peculiar functions in epithelia [86, 87] and brain development [88–91], respectively, share with wt p53 tumour suppressive abilities, which might be altered by p53 mutants [87, 92]. These mechanisms lead to the postulation of the gain-of-function (GOF) effects of p53 mutant [69, 93–95]. Hence, mutations in p53 protein sequence appear to shift the tumour suppression function. of the wild-type protein to an oncogene form in the mutant [49, 94]. Experimental evidence has however often challenged this postulation. For example, while evidence GOF phenotypes, such as growth in vitro soft-agar assays and in injected nude mice, have been shown for p53 R175H and R273H introduced in p53-null cells, p53 R172H and R270H genetically engineered mouse models did not show any alteration in survival when compared to p53-null mice. Consistently with the central role of functional (wt) p53 in the response to multiple cellular stressors, it might be not surprising that p53 mutant proteins also shift their behaviour when interacting with different microenvironmental conditions. Within this microbiome represents a major “extrinsic” factor that might influence p53 GOF; a recent work has indeed assessed the paradoxical effect that gut microbiome exerts on p53 mutant.

## Gut microbiota: a paradoxical effect on p53 mutant

**Fig. 2:**
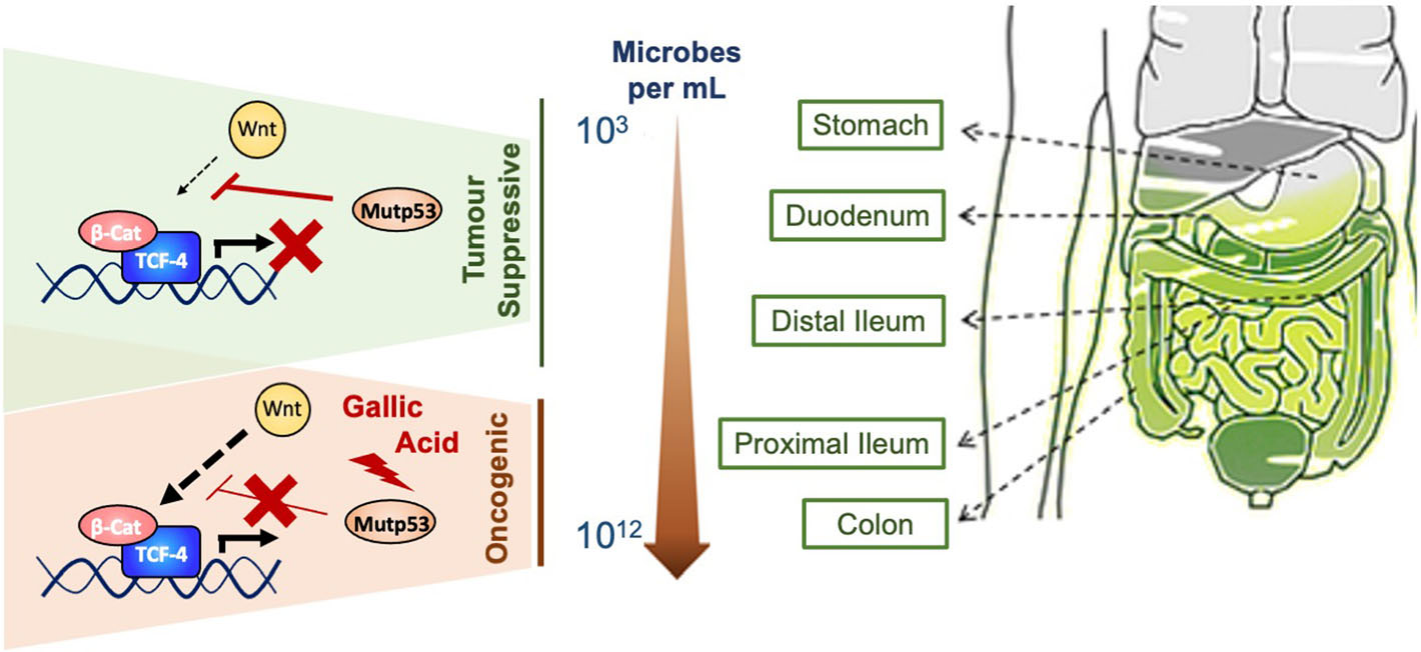
Gut microbiota switches p53 mutant from tumour suppressor to oncogene. P53 mutants (R270H and R175H) have been reported to play tumour suppressive role in the upper GI tract, by inhibiting activation of Wnt/b-Cat signalling. In the distal intestine (Colon), where gut microbiota is present in higher density, bacteria-released gallic acid appears to switch p53 mutants to oncogenic activity, abrogating its capability of opposing to Wnt signalling

Interaction between tumour suppression mechanisms and microenvironment is crucial for the cancer cell fate. This is particularly relevant in the context of p53, a tumour suppressor that largely acts a stress response protein. A recent work has addressed the role of mutant p53 in gut tumorigenesis and in particular in the interaction with the gut microbiota. Kadosh and colleagues demonstrated that mutant p53 (R172H and R270H) exerts an evident tumour-suppressive function in the proximal mouse gut, exceeding the wild-type p53 tumour suppressive abilities. Mutant p53 was shown to substantially repress WNT-driven hyperproliferation, abrogating dysplasia in CKIa^Δgut^ mice and tumorigenesis in Apc^Min/+^ mice with a mechanism that yet is not being elucidated. Conversely however, in the distal gut p53 mutant reproduced the expected oncogenic effect. From the mechanistic perspective, gallic acid produced by gut microbiota conferred to p53 mutant pro-tumorigenic function promoting malignant phenotype. Administration of gallic acid promoted reactivation of WNT-mediated TCF4 activation and promoter binding that resulted in pro-tumorigenic effects in organoids and mouse models [28] (Fig. 2).

We recently postulated that “context is everything” for the p53 mutant GOF [96] and these recent findings seem to further sustain the importance of the context on this matter. The highly unexpected nature of the findings, however open to a number of relevant questions. These clearly include the mechanisms underlying the mutant p53 tumour suppressive function, especially in the light of the demonstrated lack of binding on the wt p53 sites, but also more general questions on the reason for a high selective pressure to accumulate p53 mutations in such a wide range of human tumours. Overall the intriguing observation, that gut microbiota has a paradoxical effect on p53 mutant switching it from tumour suppressive to oncogenic protein, highlights even more the complexity and the importance of host-microbiome interaction for the disease onset and progression.

## Conclusion

Advance of the genomic technologies and computational tools in the last 20 years have significantly impacted our understanding of the genetic factors at the basis of malignancies [97–100]; more recent applications have however opened to the importance of (micro)-environmental factors on the pathogenesis of cancer. In this context microbiome has emerged as a critical player in cancer progression, not only in gut malignancies, but also more in general in distal organ-organ interactions [101]. The ability of microbiome to alter the signalling pathways of host cells is remarkable, and the recent observation of paradoxical roles in switching the function of a protein from tumour suppressive to oncogenic further underlies the potential of this multifactorial element in the pathogenesis of cancer. On the basis of these recent advance massive efforts should be placed in investigating the impact of commensal microbiome on the human health [102, 103]. This could be persuaded by investigating how recurrent antibiotics treatments and/or probiotics administration impact cancer incidence and prognosis to better define a significant connection between cancer and bacteria.

## Data Availability

Not applicable.

## References

[1] Mihaylov I, Kandula M, Krachunov M, Vassilev D (2019). A novel framework for horizontal and vertical data integration in cancer studies with application to survival time prediction models. Biol Direct.

[2] Panchin AY, Aleoshin VV, Panchin YV (2019). From tumors to species: a SCANDAL hypothesis. Biol Direct.

[3] Gouirand V, Vasseur S (2018). Fountain of youth of pancreatic cancer cells: the extracellular matrix. Cell Death Discov.

[4] Calabrese C, Davidson NR, Demircioglu D, Fonseca NA, He Y, Kahles A, Lehmann KV, Liu F, Shiraishi Y, Group PTC (2020). Genomic basis for RNA alterations in cancer. Nature.

[5] Goldman MJ, Zhang J, Fonseca NA, Cortes-Ciriano I, Xiang Q, Craft B, Pineiro-Yanez E, O'Connor BD, Bazant W, Barrera E (2020). A user guide for the online exploration and visualization of PCAWG data. Nat Commun.

[6] Gao C, Zhao D, Zhao Q, Dong D, Mu L, Zhao X, Guo M, Xu A, Fang L, Liu Q (2019). Microarray profiling and co-expression network analysis of lncRNAs and mRNAs in ovarian cancer. Cell Death Discov.

[7] Grimes T, Walker AR, Datta S, Datta S (2018). Predicting survival times for neuroblastoma patients using RNA-seq expression profiles. Biol Direct.

[8] Han Y, Ye X, Cheng J, Zhang S, Feng W, Han Z, Zhang J, Huang K (2019). Integrative analysis based on survival associated co-expression gene modules for predicting neuroblastoma patients' survival time. Biol Direct.

[9] Han Y, Ye X, Wang C, Liu Y, Zhang S, Feng W, Huang K, Zhang J (2019). Integration of molecular features with clinical information for predicting outcomes for neuroblastoma patients. Biol Direct.

[10] Pieraccioli M, Nicolai S, Pitolli C, Agostini M, Antonov A, Malewicz M, Knight RA, Raschella G, Melino G (2018). ZNF281 inhibits neuronal differentiation and is a prognostic marker for neuroblastoma. Proc Natl Acad Sci U S A.

[11] Lu JH, Zuo ZX, Wang W, Zhao Q, Qiu MZ, Luo HY, Chen ZH, Mo HY, Wang F, Yang DD (2018). A two-microRNA-based signature predicts first-line chemotherapy outcomes in advanced colorectal cancer patients. Cell Death Discov.

[12] Massari F, Di Nunno V, Guida A, Costa Silva CA, Derosa L, Mollica V, Colomba E, Brandi G, Albiges L. Addition of primary metastatic site on bone, brain, and liver to IMDC criteria in patients with metastatic renal cell carcinoma: a validation study. Clin Genitourin Cancer. 2020. 10.1016/j.clgc.2020.06.003 32694008

[13] Sun L, Ma L, Ma Y, Zhang F, Zhao C, Nie Y (2018). Insights into the role of gut microbiota in obesity: pathogenesis, mechanisms, and therapeutic perspectives. Protein Cell.

[14] Maini Rekdal V, Bess EN, Bisanz JE, Turnbaugh PJ, Balskus EP. Discovery and inhibition of an interspecies gut bacterial pathway for levodopa metabolism. Science. 2019;364. 10.1126/science.aau6323 PMC7745125 31196984

[15] Garrido-Maraver J, Celardo I, Costa AC, Lehmann S, Loh SHY, Martins LM (2019). Enhancing folic acid metabolism suppresses defects associated with loss of Drosophila mitofusin. Cell Death Dis.

[16] Hakozaki T, Richard C, Elkrief A, Hosomi Y, Benlaifaoui M, Mimpen I, Terrisse S, Derosa L, Zitvogel L, Routy B, et al. The Gut Microbiome Associates with Immune Checkpoint Inhibition Outcomes in Patients with Advanced Non-Small Cell Lung Cancer. Cancer Immunol Res. 2020. 10.1158/2326-6066.CIR-20-0196 32847937

[17] Derosa L, Routy B, Fidelle M, Iebba V, Alla L, Pasolli E, Segata N, Desnoyer A, Pietrantonio F, Ferrere G (2020). Gut Bacteria composition drives primary resistance to Cancer immunotherapy in renal cell carcinoma patients. Eur Urol.

[18] Riquelme E, Zhang Y, Zhang L, Montiel M, Zoltan M, Dong W, Quesada P, Sahin I, Chandra V, San Lucas A (2019). Tumor microbiome diversity and composition influence pancreatic Cancer outcomes. Cell.

[19] Virtue AT, McCright SJ, Wright JM, Jimenez MT, Mowel WK, Kotzin JJ, Joannas L, Basavappa MG, Spencer SP, Clark ML, et al. The gut microbiota regulates white adipose tissue inflammation and obesity via a family of microRNAs. Sci Transl Med. 2019;11. 10.1126/scitranslmed.aav1892 PMC7050429 31189717

[20] Hold GL, Garrett WS (2015). Gut microbiota. Microbiota organization--a key to understanding CRC development. Nat Rev Gastroenterol Hepatol.

[21] Walker AR, Datta S (2019). Identification of city specific important bacterial signature for the MetaSUB CAMDA challenge microbiome data. Biol Direct.

[22] Cheng WY, Wu CY, Yu J (2020). The role of gut microbiota in cancer treatment: friend or foe?. Gut.

[23] Ryan FJ (2019). Application of machine learning techniques for creating urban microbial fingerprints. Biol Direct.

[24] Smyth EC, Nilsson M, Grabsch HI, van Grieken NC, Lordick F (2020). Gastric cancer. Lancet.

[25] Kawulok J, Kawulok M, Deorowicz S (2019). Environmental metagenome classification for constructing a microbiome fingerprint. Biol Direct.

[26] L. Biancone, S. Onali, E. Calabrese, C. Petruzziello, F. Zorzi, G. Condino, G.S. Sica, F. Pallone, (2008) Non-invasive techniques for assessing postoperative recurrence in Crohn's disease. Digestive and Liver Disease 40:S265-S270. 10.1016/S1590-8658(08)60536-8 18598999

[27] P Sileri, G Sica, P Gentileschi, M Venza, A Manzelli, G Palmieri, L.G Spagnoli, G Testa, E Benedetti, A.L Gaspari, (2004) Ischemic preconditioning protects intestine from prolonged ischemia. Transplantation Proceedings 36 (2):283-285. 10.1016/j.transproceed.2004.01.078 15050134

[28] Kadosh E, Snir-Alkalay I, Venkatachalam A, May S, Lasry A, Elyada E, Zinger A, Shaham M, Vaalani G, Mernberger M, et al. The gut microbiome switches mutant p53 from tumour-suppressive to oncogenic. Nature. 2020. 10.1038/s41586-020-2541-0 PMC7116712 32728212

[29] Osmanovic D, Kessler DA, Rabin Y, Soen Y (2018). Darwinian selection of host and bacteria supports emergence of Lamarckian-like adaptation of the system as a whole. Biol Direct.

[30] Garrett WS (2015). Cancer and the microbiota. Science.

[31] Zolfo M, Asnicar F, Manghi P, Pasolli E, Tett A, Segata N (2018). Profiling microbial strains in urban environments using metagenomic sequencing data. Biol Direct.

[32] Zhu C, Miller M, Lusskin N, Mahlich Y, Wang Y, Zeng Z, Bromberg Y (2019). Fingerprinting cities: differentiating subway microbiome functionality. Biol Direct.

[33] Yamamura K, Baba Y, Nakagawa S, Mima K, Miyake K, Nakamura K, Sawayama H, Kinoshita K, Ishimoto T, Iwatsuki M (2016). Human microbiome Fusobacterium Nucleatum in esophageal Cancer tissue is associated with prognosis. Clin Cancer Res.

[34] Hsieh YY, Tung SY, Pan HY, Yen CW, Xu HW, Lin YJ, Deng YF, Hsu WT, Wu CS, Li C (2018). Increased abundance of Clostridium and Fusobacterium in gastric microbiota of patients with gastric Cancer in Taiwan. Sci Rep.

[35] Gaiser RA, Halimi A, Alkharaan H, Lu L, Davanian H, Healy K, Hugerth LW, Ateeb Z, Valente R, Fernandez Moro C (2019). Enrichment of oral microbiota in early cystic precursors to invasive pancreatic cancer. Gut.

[36] Rubinstein MR, Wang X, Liu W, Hao Y, Cai G, Han YW (2013). Fusobacterium nucleatum promotes colorectal carcinogenesis by modulating E-cadherin/beta-catenin signaling via its FadA adhesin. Cell Host Microbe.

[37] Gur C, Ibrahim Y, Isaacson B, Yamin R, Abed J, Gamliel M, Enk J, Bar-On Y, Stanietsky-Kaynan N, Coppenhagen-Glazer S (2015). Binding of the Fap2 protein of Fusobacterium nucleatum to human inhibitory receptor TIGIT protects tumors from immune cell attack. Immunity.

[38] Kaplan CW, Ma X, Paranjpe A, Jewett A, Lux R, Kinder-Haake S, Shi W (2010). Fusobacterium nucleatum outer membrane proteins Fap2 and RadD induce cell death in human lymphocytes. Infect Immun.

[39] Papapietro O, Teatero S, Thanabalasuriar A, Yuki KE, Diez E, Zhu L, Kang E, Dhillon S, Muise AM, Durocher Y (2013). R-spondin 2 signalling mediates susceptibility to fatal infectious diarrhoea. Nat Commun.

[40] Wu S, Rhee KJ, Albesiano E, Rabizadeh S, Wu X, Yen HR, Huso DL, Brancati FL, Wick E, McAllister F (2009). A human colonic commensal promotes colon tumorigenesis via activation of T helper type 17 T cell responses. Nat Med.

[41] Long X, Wong CC, Tong L, Chu ESH, Ho Szeto C, Go MYY, Coker OO, Chan AWH, Chan FKL, Sung JJY (2019). Peptostreptococcus anaerobius promotes colorectal carcinogenesis and modulates tumour immunity. Nat Microbiol.

[42] Tsoi H, Chu ESH, Zhang X, Sheng J, Nakatsu G, Ng SC, Chan AWH, Chan FKL, Sung JJY, Yu J (2017). Peptostreptococcus anaerobius induces intracellular cholesterol biosynthesis in Colon cells to induce proliferation and causes dysplasia in mice. Gastroenterology.

[43] Uribe-Herranz M, Rafail S, Beghi S, Gil-de-Gomez L, Verginadis I, Bittinger K, Pustylnikov S, Pierini S, Perales-Linares R, Blair IA (2020). Gut microbiota modulate dendritic cell antigen presentation and radiotherapy-induced antitumor immune response. J Clin Invest.

[44] Daillere R, Vetizou M, Waldschmitt N, Yamazaki T, Isnard C, Poirier-Colame V, Duong CPM, Flament C, Lepage P, Roberti MP (2016). Enterococcus hirae and Barnesiella intestinihominis facilitate cyclophosphamide-induced therapeutic Immunomodulatory effects. Immunity.

[45] Ma C, Han M, Heinrich B, Fu Q, Zhang Q, Sandhu M, Agdashian D, Terabe M, Berzofsky JA, Fako V, et al. Gut microbiome-mediated bile acid metabolism regulates liver cancer via NKT cells. Science. 2018;360. 10.1126/science.aan5931 PMC6407885 29798856

[46] Geller LT, Barzily-Rokni M, Danino T, Jonas OH, Shental N, Nejman D, Gavert N, Zwang Y, Cooper ZA, Shee K (2017). Potential role of intratumor bacteria in mediating tumor resistance to the chemotherapeutic drug gemcitabine. Science.

[47] Geller LT, Straussman R (2018). Intratumoral bacteria may elicit chemoresistance by metabolizing anticancer agents. Mol Cell Oncol.

[48] Malkin D, Li FP, Strong LC, Fraumeni JF, Nelson CE, Kim DH, Kassel J, Gryka MA, Bischoff FZ, Tainsky MA (1990). Germ line p53 mutations in a familial syndrome of breast cancer, sarcomas, and other neoplasms. Science.

[49] Pitolli C, Wang Y, Mancini M, Shi Y, Melino G, Amelio I. Do mutations turn p53 into an oncogene? Int J Mol Sci. 2019;20. 10.3390/ijms20246241 PMC6940991 31835684

[50] Pitolli C, Wang Y, Candi E, Shi Y, Melino G, Amelio I. p53-mediated tumor suppression: DNA-damage response and alternative mechanisms. Cancers (Basel). 2019;11. 10.3390/cancers11121983 PMC6966539 31835405

[51] Amelio I, Melino G (2015). The p53 family and the hypoxia-inducible factors (HIFs): determinants of cancer progression. Trends Biochem Sci.

[52] Catizone AN, Good CR, Alexander KA, Berger SL, Sammons MA (2019). Comparison of genotoxic versus nongenotoxic stabilization of p53 provides insight into parallel stress-responsive transcriptional networks.

[53] Kaiser AM, Attardi LD (2018). Deconstructing networks of p53-mediated tumor suppression in vivo. Cell Death Differ.

[54] Castrogiovanni C, Waterschoot B, De Backer O, Dumont P (2018). Serine 392 phosphorylation modulates p53 mitochondrial translocation and transcription-independent apoptosis. Cell Death Differ.

[55] el-Deiry WS, Tokino T, Velculescu VE, Levy DB, Parsons R, Trent JM, Lin D, Mercer WE, Kinzler KW, Vogelstein B (1993). WAF1, a potential mediator of p53 tumor suppression. Cell.

[56] Harper JW, Adami GR, Wei N, Keyomarsi K, Elledge SJ (1993). The p21 Cdk-interacting protein Cip1 is a potent inhibitor of G1 cyclin-dependent kinases. Cell.

[57] Engeland K (2018). Cell cycle arrest through indirect transcriptional repression by p53: I have a DREAM. Cell Death Differ.

[58] Vazquez A, Bond EE, Levine AJ, Bond GL (2008). The genetics of the p53 pathway, apoptosis and cancer therapy. Nat Rev Drug Discov.

[59] Ho DH, Seol W, Son I (2019). Upregulation of the p53-p21 pathway by G2019S LRRK2 contributes to the cellular senescence and accumulation of alpha-synuclein.

[60] Riley T, Sontag E, Chen P, Levine A (2008). Transcriptional control of human p53-regulated genes. Nat Rev Mol Cell Biol.

[61] Lin RW, Ho CJ, Chen HW, Pao YH, Chen LE, Yang MC, Huang SB, Wang S, Chen CH, Wang C. P53 enhances apoptosis induced by doxorubicin only under conditions of severe DNA damage. 2018;17:2175–86. 10.1080/15384101.2018.1520565 PMC6226221 30198376

[62] Sullivan KD, Galbraith MD, Andrysik Z, Espinosa JM (2018). Mechanisms of transcriptional regulation by p53. Cell Death Differ.

[63] Arena G, Riscal R, Linares LK, Le Cam L (2018). MDM2 controls gene expression independently of p53 in both normal and cancer cells. Cell Death Differ.

[64] Williams AB, Schumacher B. p53 in the DNA-damage-repair process. Cold Spring Harb Perspect Med. 2016;6. 10.1101/cshperspect.a026070 PMC4852800 27048304

[65] Lim Y, De Bellis D, Dorstyn L, Kumar S (2018). p53 accumulation following cytokinesis failure in the absence of caspase-2. Cell Death Differ.

[66] Sankunny M, Eng C (2018). KLLN-mediated DNA damage-induced apoptosis is associated with regulation of p53 phosphorylation and acetylation in breast cancer cells. Cell Death Discov.

[67] Hunger A, Medrano RF, Zanatta DB, Del Valle PR, Merkel CA, Salles TA, Ferrari DG, Furuya TK, Bustos SO, de Freitas SR (2017). Reestablishment of p53/Arf and interferon-beta pathways mediated by a novel adenoviral vector potentiates antiviral response and immunogenic cell death. Cell Death Discov.

[68] Wu D, Prives C (2018). Relevance of the p53-MDM2 axis to aging. Cell Death Differ.

[69] Mantovani F, Collavin L, Del Sal G (2019). Mutant p53 as a guardian of the cancer cell. Cell Death Differ.

[70] Amelio I (2019). How mutant p53 empowers Foxh1 fostering leukaemogenesis?. Cell Death Discov.

[71] Klimovich B, Stiewe T, Timofeev O. Inactivation of Mdm2 restores apoptosis proficiency of cooperativity mutant p53 in vivo. 2020; 19:109–23. 10.1080/15384101.2019.1693748 PMC6927723 31749402

[72] Adorno M, Cordenonsi M, Montagner M, Dupont S, Wong C, Hann B, Solari A, Bobisse S, Rondina MB, Guzzardo V (2009). A mutant-p53/Smad complex opposes p63 to empower TGFbeta-induced metastasis. Cell.

[73] Muller PA, Caswell PT, Doyle B, Iwanicki MP, Tan EH, Karim S, Lukashchuk N, Gillespie DA, Ludwig RL, Gosselin P (2009). Mutant p53 drives invasion by promoting integrin recycling. Cell.

[74] Celardo I, Grespi F, Antonov A, Bernassola F, Garabadgiu AV, Melino G, Amelio I (2013). Caspase-1 is a novel target of p63 in tumor suppression. Cell Death Dis.

[75] Celardo I, Antonov A, Amelio I, Annicchiarico-Petruzzelli M, Melino G (2014). p63 transcriptionally regulates the expression of matrix metallopeptidase 13. Oncotarget.

[76] Candi E, Terrinoni A, Rufini A, Chikh A, Lena AM, Suzuki Y, Sayan BS, Knight RA, Melino G (2006). p63 is upstream of IKK alpha in epidermal development. J Cell Sci.

[77] Amelio I, Antonov AA, Catani MV, Massoud R, Bernassola F, Knight RA, Melino G, Rufini A (2014). TAp73 promotes anabolism. Oncotarget.

[78] Nemajerova A, Amelio I, Gebel J, Dotsch V, Melino G, Moll UM (2018). Non-oncogenic roles of TAp73: from multiciliogenesis to metabolism. Cell Death Differ.

[79] Lena AM, Cipollone R, Amelio I, Catani MV, Ramadan S, Browne G, Melino G, Candi E (2010). Skn-1a/Oct-11 and DeltaNp63alpha exert antagonizing effects on human keratin expression. Biochem Biophys Res Commun.

[80] Weissmueller S, Manchado E, Saborowski M, Morris JP, Wagenblast E, Davis CA, Moon SH, Pfister NT, Tschaharganeh DF, Kitzing T (2014). Mutant p53 drives pancreatic cancer metastasis through cell-autonomous PDGF receptor beta signaling. Cell.

[81] Amelio I, Inoue S, Markert EK, Levine AJ, Knight RA, Mak TW, Melino G (2015). TAp73 opposes tumor angiogenesis by promoting hypoxia-inducible factor 1alpha degradation. Proc Natl Acad Sci U S A.

[82] Amelio I, Mancini M, Petrova V, Cairns RA, Vikhreva P, Nicolai S, Marini A, Antonov AA, Le Quesne J, Baena Acevedo JD (2018). p53 mutants cooperate with HIF-1 in transcriptional regulation of extracellular matrix components to promote tumor progression. Proc Natl Acad Sci U S A.

[83] Duffy MJ, Synnott NC, O'Grady S, Crown J. Targeting p53 for the treatment of cancer. Semin Cancer Biol. 2020. 10.1016/j.semcancer.2020.07.005 32741700

[84] Duffy MJ (2020). Biomarkers for prostate cancer: prostate-specific antigen and beyond. Clin Chem Lab Med.

[85] Insabato L, Amelio I, Quarto M, Zannetti A, Tolino F, de Mauro G, Cerchia L, Riccio P, Baumhoer D, Condorelli G (2009). Elevated expression of the tyrosine phosphatase SHP-1 defines a subset of high-grade breast tumors. Oncology.

[86] Candi E, Amelio I, Agostini M, Melino G (2015). MicroRNAs and p63 in epithelial stemness. Cell Death Differ.

[87] Levrero M, De Laurenzi V, Costanzo A, Gong J, Melino G, Wang JY (1999). Structure, function and regulation of p63 and p73. Cell Death Differ.

[88] Billon N, Terrinoni A, Jolicoeur C, McCarthy A, Richardson WD, Melino G, Raff M (2004). Roles for p53 and p73 during oligodendrocyte development. Development.

[89] Tomasini R, Tsuchihara K, Wilhelm M, Fujitani M, Rufini A, Cheung CC, Khan F, Itie-Youten A, Wakeham A, Tsao MS (2008). TAp73 knockout shows genomic instability with infertility and tumor suppressor functions. Genes Dev.

[90] Rossi M, Aqeilan RI, Neale M, Candi E, Salomoni P, Knight RA, Croce CM, Melino G (2006). The E3 ubiquitin ligase itch controls the protein stability of p63. Proc Natl Acad Sci U S A.

[91] Tomasini R, Tsuchihara K, Tsuda C, Lau SK, Wilhelm M, Rufini A, Tsao MS, Iovanna JL, Jurisicova A, Melino G (2009). TAp73 regulates the spindle assembly checkpoint by modulating BubR1 activity. Proc Natl Acad Sci U S A.

[92] Candi E, Agostini M, Melino G, Bernassola F (2014). How the TP53 family proteins TP63 and TP73 contribute to tumorigenesis: regulators and effectors. Hum Mutat.

[93] Huang Y, Liu N, Liu J, Liu Y, Zhang C, Long S, Luo G, Zhang L, Zhang Y (2019). Mutant p53 drives cancer chemotherapy resistance due to loss of function on activating transcription of PUMA.

[94] Tavana O, Sun H, Gu W. Targeting HAUSP in both p53 wildtype and p53-mutant tumors. 2018; 17:823–8. 10.1080/15384101.2018.1456293 PMC6056219 29616860

[95] Parrales A, Thoenen E, Iwakuma T (2018). The interplay between mutant p53 and the mevalonate pathway. Cell Death Differ.

[96] Amelio I, Melino G (2020). Context is everything: extrinsic signalling and gain-of-function p53 mutants. Cell Death Discov.

[97] Francescatto M, Chierici M, Rezvan Dezfooli S, Zandona A, Jurman G, Furlanello C (2018). Multi-omics integration for neuroblastoma clinical endpoint prediction. Biol Direct.

[98] Kim SY, Jeong HH, Kim J, Moon JH, Sohn KA (2019). Robust pathway-based multi-omics data integration using directed random walks for survival prediction in multiple cancer studies. Biol Direct.

[99] Mandal P, Saha SS, Sen S, Bhattacharya A, Bhattacharya NP, Bucha S, Sinha M, Chowdhury RR, Mondal NR, Chakravarty B (2019). Cervical cancer subtypes harbouring integrated and/or episomal HPV16 portray distinct molecular phenotypes based on transcriptome profiling of mRNAs and miRNAs. Cell Death Discov.

[100] Amelio I, Tsvetkov PO, Knight RA, Lisitsa A, Melino G, Antonov AV (2016). SynTarget: an online tool to test the synergetic effect of genes on survival outcome in cancer. Cell Death Differ.

[101] Caputo A, Fournier PE, Raoult D (2019). Genome and pan-genome analysis to classify emerging bacteria. Biol Direct.

[102] Gerner SM, Rattei T, Graf AB (2018). Assessment of urban microbiome assemblies with the help of targeted in silico gold standards. Biol Direct.

[103] Walker AR, Grimes TL, Datta S, Datta S (2018). Unraveling bacterial fingerprints of city subways from microbiome 16S gene profiles. Biol Direct.

